# Effect of chewing betel nut on the gut microbiota of *Hainanese*

**DOI:** 10.1371/journal.pone.0258489

**Published:** 2021-10-14

**Authors:** Li Ying, Yunjia Yang, Jun Zhou, Hairong Huang, Guankui Du

**Affiliations:** 1 The Key Laboratory of Molecular Biology, Hainan Medical University, Haikou, China; 2 Haikou Customs, District P. R. China, Haikou, P. R. China; 3 School of Public Health, Hainan Medical University, Haikou, China; 4 Biotechnology Major, Hainan Medical University, Haikou, China; 5 Department of Biochemistry and Molecular Biology, Hainan Medical University, Haikou, China; University of Utah, UNITED STATES

## Abstract

Betel nut chewing (BNC) is prevalent in South Asia and Southeast Asia. BNC can affect host health by modulating the gut microbiota. The aim of this study is to evaluate the effect of BNC on the gut microbiota of the host. Feces samples were obtained from 34 BNC individuals from Ledong and Lingshui, Hainan, China. The microbiota was analyzed by 16S rRNA gene sequencing. BNC decreased the microbial α-diversity. Firmicutes, Bacteroidetes, Actinobacteria, and Proteobacteria were the predominant phyla, accounting for 99.35% of the BNC group. The Firmicutes-to-Bacteroidetes ratio was significantly increased in the BNC group compared to a control group. The abundances of the families Aerococcaceae, Neisseriaceae, Moraxellaceae, Porphyromonadaceae, and Planococcaceae were decreased in the BNC/BNC_Male/BNC_Female groups compared to the control group, whereas the abundances of Coriobacteriaceae, Streptococcaceae, Micrococcaceae, Xanthomonadaceae, Coxiellaceae, Nocardioidaceae, Rhodobacteraceae, and Succinivibrionaceae were increased. In general, the gut microbiome profiles suggest that BNC may have positive effects, such as an increase in the abundance of beneficial microbes and a reduction in the abundance of disease-related microbes. However, BNC may also produce an increase in the abundance of disease-related microbes. Therefore, extraction of prebiotic components could increase the beneficial value of betel nut.

## 1. Introduction

Betel nut chewing (BNC) is prevalent in South Asia and Southeast Asia [1, 2]. Betel nut is the fourth most addictive consumer product globally, surpassed only by tobacco, alcohol, and coffee [3]. Betel nut is consumed widely by approximately 600 million people worldwide [3]. The southern part of China, especially Hainan, is the main production and consumption area for betel nut [4].

Betel nut is a traditional Chinese medicine used to treat parasitic diseases, various gastrointestinal disorders (abdominal distension, abdominal pain, dyspepsia, and diarrhea), and jaundice [5]. Modern pharmacology shows that betel nut has various pharmacological properties, including antifatigue, antioxidant, antibacterial, antifungal, antihypertensive, antidepressant, anti-inflammatory, analgesic, and antiallergic properties; betel nut also promotes digestive function, inhibits platelet aggregation, and regulates blood glucose and blood lipids [6]. However, many studies have shown that betel nut may be harmful to health, including increasing the risk of cancers, obesity, cardiovascular disease, diabetes, and chronic kidney disease [7–9]. Thus, it is necessary to consider the effects of BNC on health from multiple perspectives, such as by investigating gut microbiota.

Hundreds of millions of bacteria colonize the human intestine [10]. Gut microbiota can affect body weight, digestive ability, disease occurrence, and drug response [10]. Gut microbiota can affect host health in multiple ways, such as by synthesizing a variety of vitamins necessary for human growth and development, using protein residues to synthesize essential amino acids, participating in carbohydrate and protein metabolism, and promoting the absorption of iron, magnesium, zinc and other mineral elements [11, 12]. Moreover, gut microbiota are affected by various factors, such as the intestinal microenvironment, mental state, and diet [13]. Diet is one of the most critical factors [14]. Therefore, we hypothesized that BNC plays a role in modulating gut microbiota and thereby affects host health.

## 2. Materials and methods

### 2.1. Subjects

Participants were recruited from Ledong and Lingshui, Hainan. Fecal samples were collected from 18–60 age adults who self-reported physical health during the six months between March and September 2020. A total of 34 subjects who chewed betel nuts every day and 37 subjects who have never chewed betel nuts were selected from this cohort. The individuals were matched by age (within three years), sex, and body mass index (BMI). No individual had used antibiotics within two months of enrollment in the study. Individuals with diabetes, hypertension, diarrhea and other diseases were also excluded from the study cohort. The values of the parameters of age, sex, BMI, history of smoking, alcohol intake, betel nut intake, and laboratory results were collected and compared. Ethical approval for the microbiota studies was provided by Hainan Medical University (HNMU 2020-02-09-01). All participants consented to the study, and the potential consequences of the study were explained in detail to the participants. This study complied with all applicable institutional and government regulations on the ethical use of human volunteers.

The subjects were grouped by gender (Table 1). The individuals in the BNC group included 17 males (the BNC_Male group) and 17 females (the BNC_Female group). The control (Ctr) group included 18 males (Ctr_Male) and 19 females (Ctr_Female).

**Table 1:** Demographic and anthropometric characteristics.

	BNC		Control	
	Male	Female	Male	Female
Number	17	17	18	19
Age	31.47±11.19	46.52±9.83	30.94±12.85	40.57±12.72
BMI	22.98±3.16	22.18±5.25	22.57±1.65	21.04±2.07
Frequency	Daily		Never	
BN number/DAY	5.23±1.85	5.35±1.74	Not Applicable	Not Applicable
Duration (year)	More than four years		Not Applicable	
Smoking (%)	82.35	11.76	55.55	0
Drinking (%)	76.47	35.29	61.53	10.52

### 2.2. Fecal samples and DNA extraction

The fecal samples were frozen at -20°C immediately after defecation and stored at -70°C for less than 24 hours. Within one month, DNA was extracted from the fecal samples using the QIAamp DNA Stool Minikit (Qiagen, Hilden, Germany) according to the manufacturer’s instructions.

### 2.3. Microbiota analyses by 16S rRNA gene sequencing

According to the manufacturer’s instructions, total fecal microbiota DNA was extracted by a QIAamp DNA Stool Minikit (Qiagen, Hilden, Germany). The V3-V4 variable region (~450 bp) of the 16S rRNA was amplified using the forward primer 341F (5’-CCTACGGGNGGCWGCAG-3’) and the reverse primer 802R (5’-TACNVGGGTATCTAATCC-3’) attached to a barcode [5′-A-adapter-N (10) + 16S primer-3′]. PCR was performed as follows: 10 ng of the purified DNA, 15 μl of Phusion^®^ High-Fidelity PCR Master Mix (New England Biolabs), 200 nmol/L of the forward and reverse primers, and nuclease-free water were mixed and made up to a final volume of 25 μl. The PCR cycling conditions consisted of initial denaturation for 5 min at 95°C, followed by 30 cycles of 30 s at 95°C, 30 s at 50°C, and 5 min at 72°C. The PCR products were detected by 1% agarose gel electrophoresis and purification with a nucleic acid purification kit (Agencourt AMPure XP) and used for Illumina HiSeq/MiSeq platform sequencing to a depth of at least 30,000 reads per sample.

After MiSeq sequencing, the paired-end (PE) sequence data were subjected to quality control processing, and high-quality FASTA data were finally obtained. The quality control of fastq data was performed by Trimmomatic (v0.36) and PEAR (v0.9.6). A sliding window strategy was used in Trimmomatic. The window size was set to 50 bp, the average quality value was 20, and the minimum reserved sequence length was 120. Pear was used to remove the sequences with N. FLASH (v1.20), and PEAR was used to merge the two end sequences based on the PE overlap relationship. The minimum overlap was set at 10 bp, and the mismatch rate was 0.1 to obtain the FASTA sequence. VSEARCH (v2.7.1) was utilized to remove chimeras by the UCHIME method. After removal of the barcode and primer and splicing, raw tags were obtained and then removed from the chimeras and short sequences to obtain high-quality-sequence clean tags.

The sequences were classified by similarity into operational taxonomic units (OTUs) using the Greengenes database and the UPARSE algorithm. A bioinformatics analysis of OTUs was performed at a 97% similarity level. The RDP classifier algorithm was used to compare and analyze the OTU representative sequences. The community species information was annotated at each level (phylum, class, order, family, genus, and species).

The diversity of a microbial community can be measured using the Chao1, Simpson, and Shannon indexes. Chao1 is a species richness index and was used to estimate the number of OTUs in the community. The following formula was used: Chao1 = OBS+n1(n1-1)/2(n2+1), where Chao1 and OBS were the estimated and observed number of OTUs, respectively; and n1 and n2 were the number of OTUs with only one and only two sequences, respectively. The Simpson index was obtained as a 1-D value. The following formula was used: 1-D = 1−∑(Ni/N)^2. The D value was the total number of individuals (Ni) of a particular species found divided by the total number of individuals found (N). The following Shannon’s formula was used: H = −∑(Pi) (ln Pi), where Pi was the proportion of individuals belonging to species i in the sample.

### 2.4. Ethics statement

Ethical approval for the microbiota studies was provided by Hainan Medical University (HNMU 2020-02-09-01). The data and sample collector introduced the purpose of the study to the participants. Oral informed consent was obtained from all participants. This study complied with all applicable institutional and government regulations on the ethical use of human volunteers. To ensure confidentiality, the names and detailed addresses of the participants were not recorded during the data collection process.

### 2.5. Statistics

A partial least squares discrimination analysis (PLS-DA) was carried out to study similarities or differences in the microbial community composition. Metastats analysis and LDA effect size analysis were carried out to determine the difference between the two study groups. First, ANOVA was used to detect species with significant differences in abundance among the different groups using a threshold of 0.05. The Wilcoxon rank-sum test was used to analyze the significantly different species obtained in the previous step using a threshold of 0.05. Finally, linear discriminant analysis (LDA) was used to evaluate species’ influence with significant differences (i.e., in terms of the LDA score) using a threshold of 3.

All data were presented as the mean±SEM. A statistical analysis of physiological and biochemical data was conducted using GraphPad Prism version 7.0 (GraphPad Software, San Diego, CA).

## 3. Results

Seventy-one subjects (34 in the BNC group and 37 in the control group) were analyzed. The two groups were comparable in terms of age, sex, BMI, betel nut intake frequency and quantity, history of smoking, and alcohol intake (Table 1). Sequencing of 16S bacterial RNA retrieved an overall number of 5,292,428 reads after filtering, clustered in 1,282 operational taxonomic units.

### 3.1. The effect of BNC on gut microbial diversity and composition

**Fig 1:**
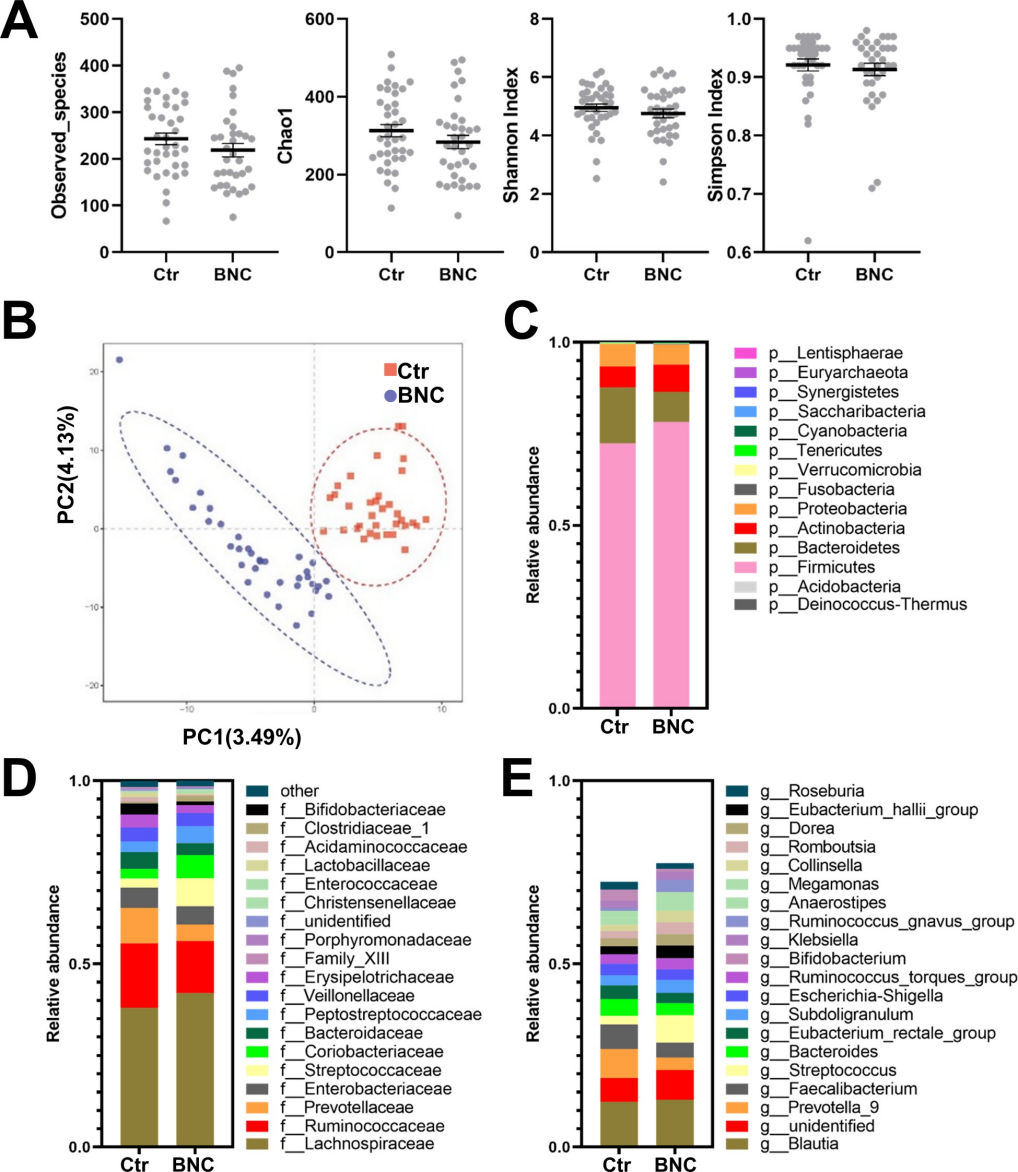
The effect of BNC on microbial diversity and composition. BNC did not affect the (A) α-diversity in terms of the observed_species and the Chao1, Shannon, and Simpson indexes. The BNC and Ctr groups could be separated on the PLS-DA plot (B). BNC affected the (C) phylum-level, (D) family-level, and (E) genus-level taxonomic distributions of the feces of the microbial communities.

The α-diversity analysis showed that the observed_species and the Chao1, Shannon, and Simpson indexes of the BNC group were lower than those of the Ctr group (P>0.05) (Fig 1A). The BNC and Ctr groups could be separated on the PLS-DA plot (Fig 1B).

The community structure was compared among groups. Firmicutes, Bacteroidetes, Actinobacteria, and Proteobacteria were the predominant (99.35%) phyla in the BNC group (Fig 1C). The abundance of Bacteroidetes was significantly lower in the BNC group compared to the Ctr group (S1 Table). Moreover, the Firmicutes-to-Bacteroidetes ratio was significantly higher in the BNC group (9.52) than the Ctr group (4.75).

At the family level, Lachnospiraceae, Ruminococcaceae, Streptococcaceae, Coriobacteriaceae, Enterobacteriaceae, Peptostreptococcaceae, Prevotellaceae, Veillonellaceae, Bacteroidaceae, and Erysipelotrichaceae were the 10 most abundant (93.37%) fecal microbiota BNC group (Fig 1D). Significant changes for 20 families were identified for the BNC group compared to the Ctr group (S1 Table). That is, compared to the Ctr group, the BNC group exhibited a significant increase in abundance for 14 families and a significant decrease in abundance for six families.

**Fig 2:**
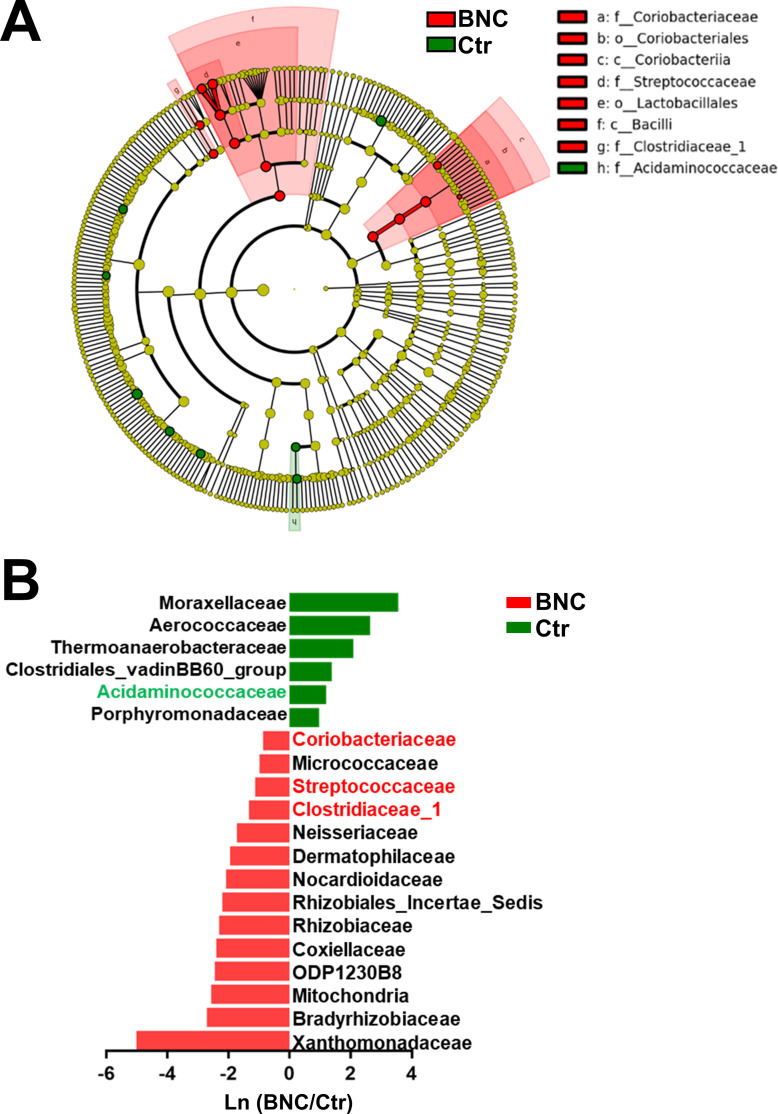
Characteristic microbes affected by BNC. (A) Cladogram of the microbial taxa associated with BNC. (B) Metastats analysis.

At the genus level, Blautia, Streptococcus, Faecalibacterium, Ruminococcus_torques_group, Ruminococcus_gnavus_group, Subdoligranulum, Prevotella, Bacteroides, Romboutsia, and Collinsella were the 10 most abundant (56.03%) fecal microbiota in the BNC group (Fig 2). The abundance of 11 genera were significantly increased and the abundance of 11 other genera were significantly reduced (S1 Table) for the BNC group compared to the Ctr group.

The Metastats analysis was combined with the linear discriminant analysis effect size (LEfSe) method to identify the specific microbial taxa associated with BNC. Compared with the Ctr group, the abundances of Coriobacteriaceae, Streptococcaceae, and Clostridiaceae_1 were significantly increased in the BNC group, whereas that of Acidaminococcaceae was significantly decreased (Fig 2).

### 3.2. Effect of BNC on female gut microbiota

**Fig 3:**
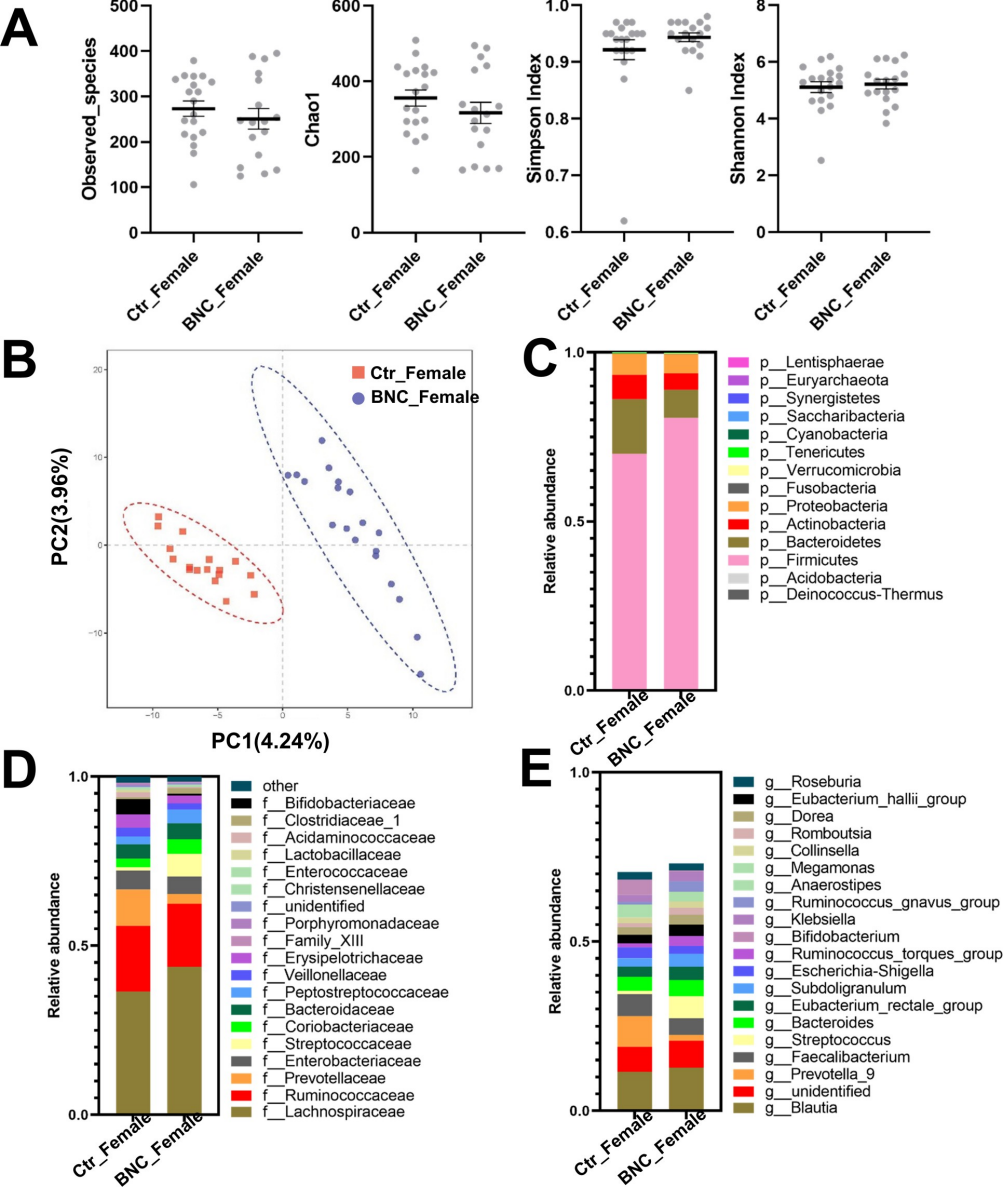
The effect of BNC on the microbial diversity and composition of females. BNC did not affect the (A) α-diversity in terms of the observed_species and Chao1, Shannon, and Simpson indexes. The BNC and Ctr groups could be separated on the PLS-DA plot (B). BNC affected the (C) phylum-level, (D) family-level, and (E) genus-level taxonomic distributions of the feces of the microbial communities.

**Fig 4:**
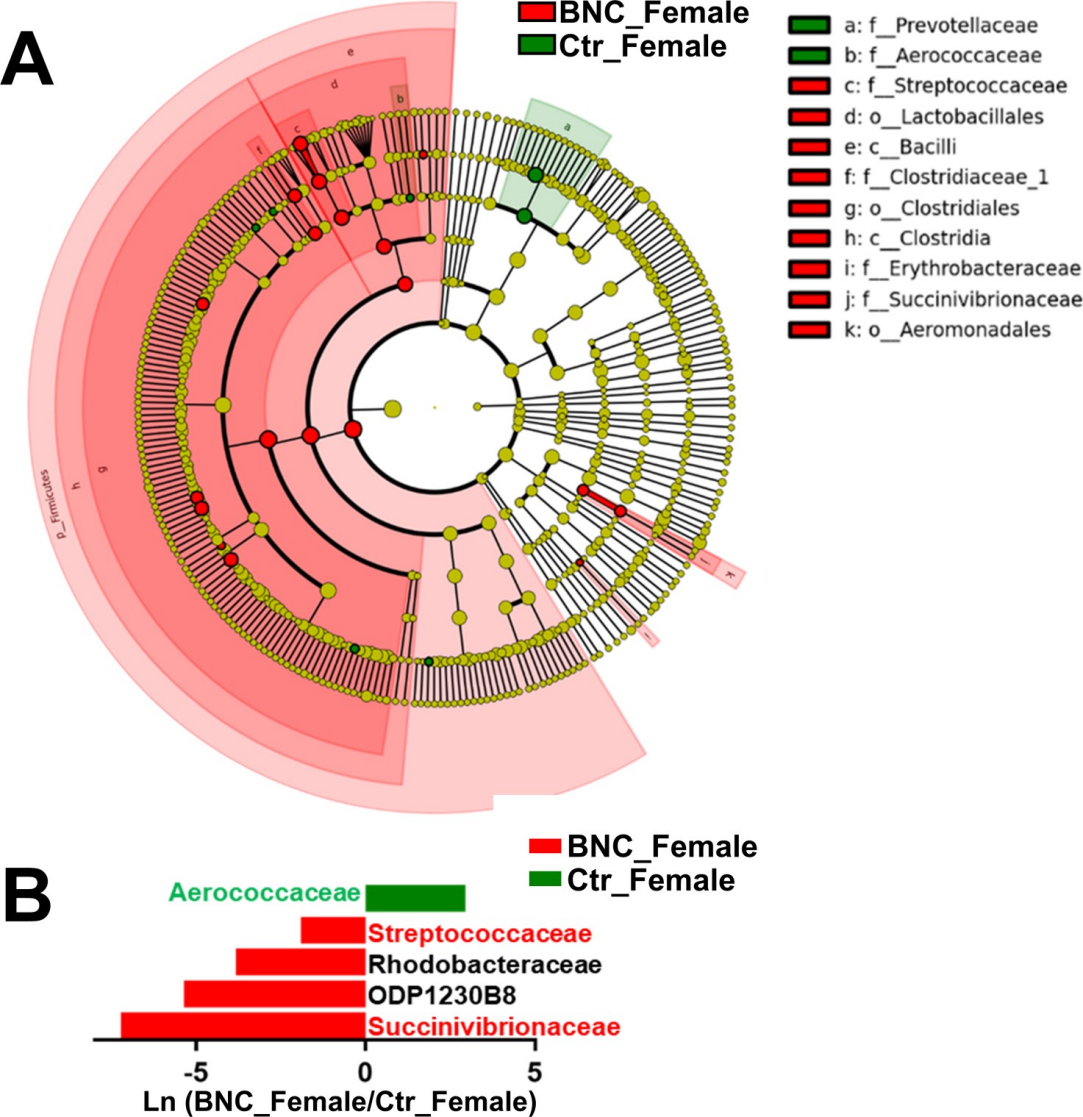
Characteristic microbes affected in the Ctr_Female group. (A) Cladogram of the microbial taxa associated with BNC. (B) Metastats analysis.

The α-diversity analysis showed no significant alteration in the BNC_Female group compared with the Ctr_Female group (Fig 3A). The BNC_Female group and Ctr_Female group could be separated by PLS-DA analysis (Fig 3B). At the phylum level, the abundances of Firmicutes and Euryarchaeota were significantly increased in the BNC_Female group (Fig 3C and S2 Table) compared to the Ctr_Female group. The Firmicutes-to-Bacteroidetes ratio increased dramatically in the BNC_Female group (9.67) compared with the Ctr_Female group (4.35). Moreover, a significant change was identified for five families in the BNC_Female group (Fig 3D) compared to the Ctr_Female group. The abundances of 4 families were significantly increased and the abundance of one family was significantly decreased (S2 Table) in the BNC_Female group compared with the Ctr_Female group. Compared to the Ctr_Female group, six genera were significantly increased in abundance at the genus level and three genes were significantly reduced in abundance in the BNC_Female group (S2 Table). The results of the Metastats and LEfSe combined analysis showed that the abundance of Streptococcaceae and Succinivibrionaceae significantly increased and the abundance of Aerococcaceae significantly decreased in the BNC_Female group compared to the Ctr_Female group (Fig 4).

### 3.3. Effect of BNC on male gut microbiota

**Fig 5:**
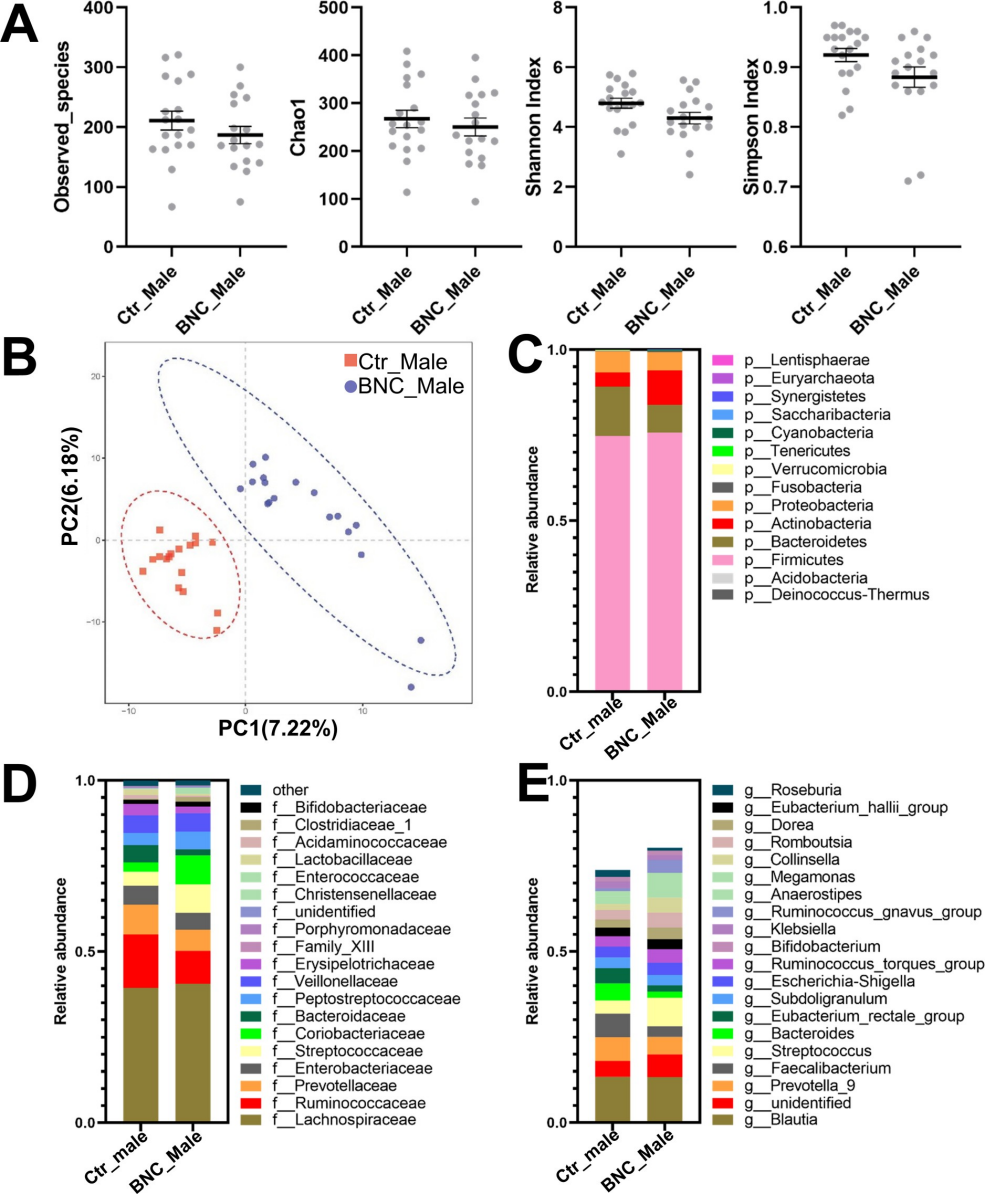
The effect of BNC on the microbial diversity and composition of males. BNC did not affect the (A) α-diversity in terms of the observed_species and the Chao1, Shannon, and Simpson indexes. The BNC and Ctr groups could be separated on the PLS-DA plot (B). BNC affected the (C) phylum-level, (D) family-level, and (E) genus-level taxonomic distributions of the feces of the microbial communities.

**Fig 6:**
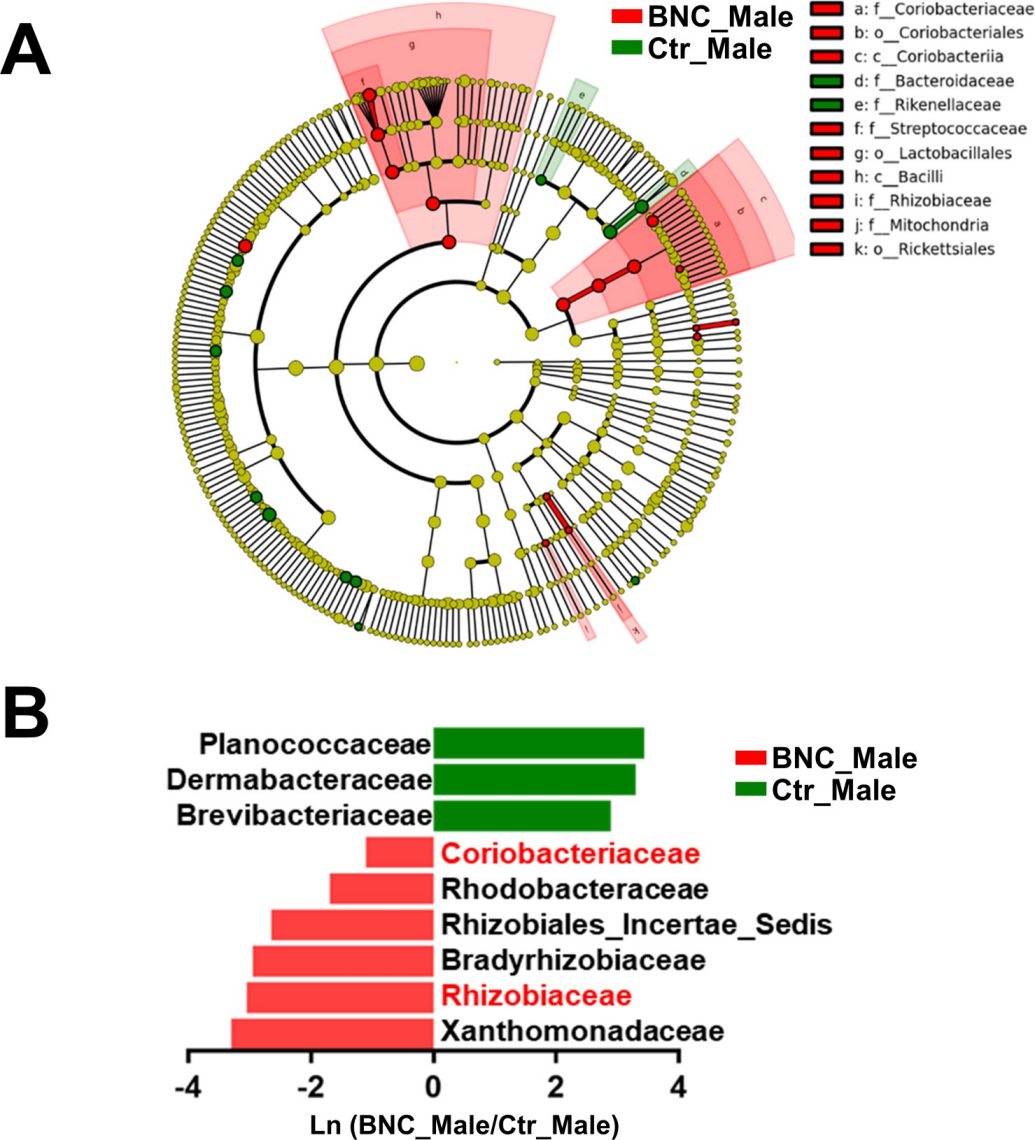
Characteristic microbes affected in the Ctr_Male group. (A) Cladogram of the microbial taxa associated with BNC. (B) Metastats analysis.

The α-diversity analysis showed no significant alteration in the BNC_Male group compared with the Ctr_Male group (Fig 5A). The BNC_Male and Ctr_Male groups could be separated by PLS-DA analysis (Fig 5B). At the phylum level, there was a higher proportion of Actinobacteria and Cyanobacteria in the BNC_Male group than the Ctr_Male group (Fig 5C and S3 Table). The Firmicutes-to-Bacteroidetes ratio was significantly increased in the BNC_Male group (9.39) compared with the Ctr_Male group (5.20). Significant changes were identified for nine families in the BNC_Male group compared to the Ctr_Male group (Fig 5D). That is, the abundances of six families were significantly increased and the abundances of three families were significantly decreased in the BNC_Male group compared to the Ctr_Male group (S3 Table). The abundances of 4 genera were significantly increased and the abundances of 12 other genera were significantly decreased in the BNC_Male group compared to the Ctr_Male group (S3 Table). The Metastats and LEfSe combined analysis showed that the abundances of Coriobacteriaceae and Rhizobiaceae significantly increased in the BNC_Male group compared with the Ctr_Male group (Fig 6).

### 3.4. The alteration of microbial function

**Fig 7:**
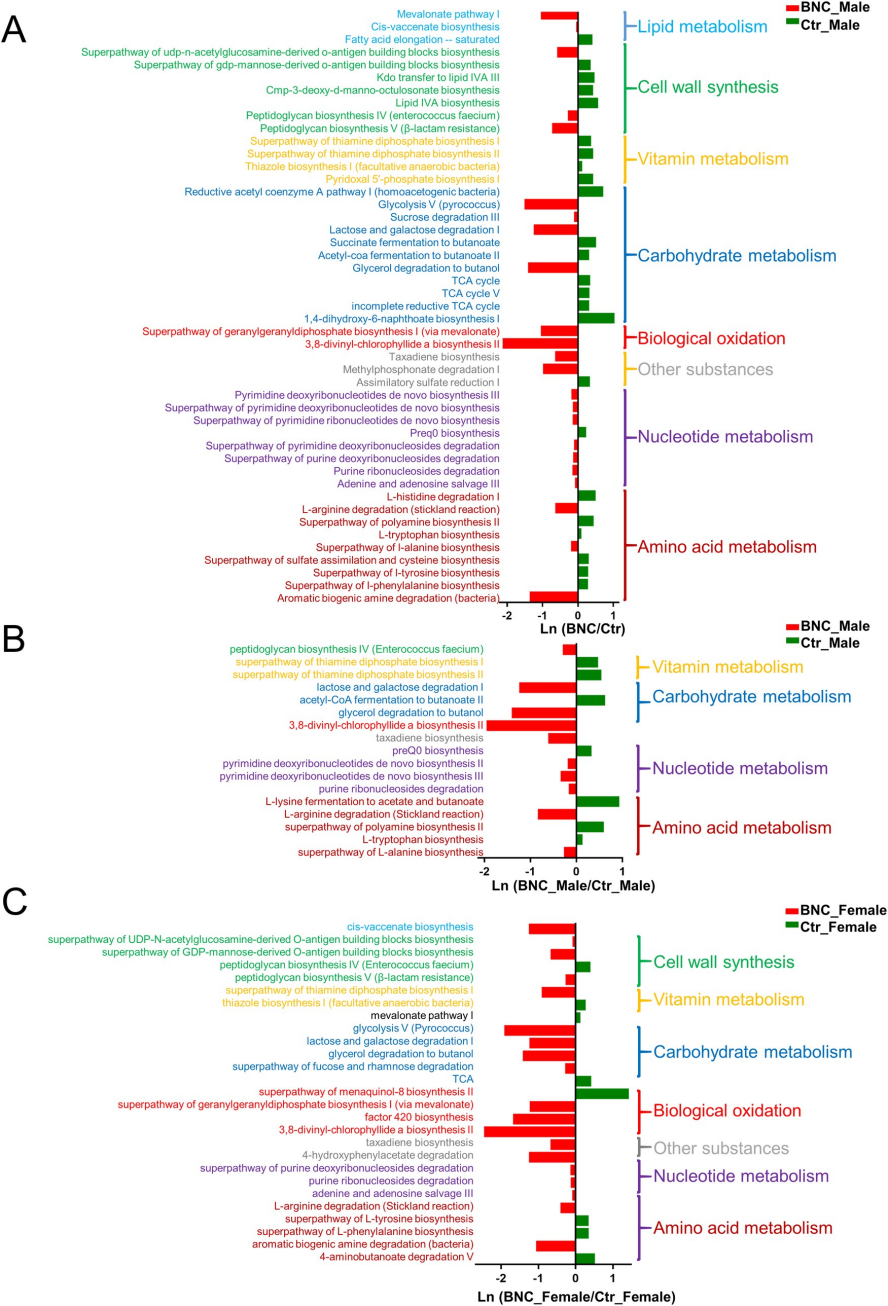
The effect of BNC on microbial function. The significant effect of BNC on microbial metabolism pathways in (A) all individuals, (B) female, and (C) male individuals.

The alteration of microbial function was identified by using picrust2 to predict the metabolic pathways. Forty-seven, 17, and 27 pathways were identified in the BNC, BNC_Male, and BNC_Female groups, respectively (Fig 7). In total, 54 pathways were involved in BNC, whereas eight pathways (L-arginine degradation, lactose and galactose degradation I, purine ribonucleoside degradation, 3,8-divinyl-chlorophyllide a biosynthesis II, peptidoglycan biosynthesis IV, glycerol degradation to butanol, taxadiene biosynthesis, and the superpathway of thiamine diphosphate biosynthesis I) were found for the three groups.

In general, these pathways are involved in the metabolism of various substances (Fig 7). The number of lipid metabolism pathways, such as mevalonate pathway I and cisvaccenate biosynthesis, were significantly increased for the BNC group compared to the Ctr group. The synthesis of vitamins, especially vitamin B1, was significantly decreased in the BNC group compared to the Ctr group. Compared to the Ctr group, in the BNC group, the number of amino acid synthesis pathways (such as L-histidine degradation I and the superpathways of polyamine biosynthesis II, L-tryptophan biosynthesis, sulfate assimilation and cysteine biosynthesis, L-tyrosine biosynthesis, and L-phenylalanine biosynthesis) were significantly decreased, whereas the number of pathways for amino acid degradation (L-arginine degradation (the Stickland reaction) and aromatic biogenic amine degradation (bacteria)) were significantly increased. In addition, the number of carbohydrate degradation pathways (glycolysis V (pyrococcus), sucrose degradation III, lactose and galactose degradation I, and glycerol degradation to butanol) were significantly increased for the BNC group compared to the Ctr group, whereas the number of TCA cycle pathways (TCA, TCA cycle V and incomplete reductive TCA) were significantly decreased. Moreover, the number of nucleotide metabolism pathways, for both biosynthesis and degradation, were significantly increased for the BNC group compared to the Ctr group.

## 4. Discussion

BNC is a prevalent behavior in Hainan. Among the considered factors, long-term diet quality has a predominant impact on gut microbiota [15]. The active component of betel nut enters the intestine in the juice produced by chewing betel nut and affects the intestinal microenvironment. Therefore, chewing betel nut can affect the diversity and composition of gut microbiota. In this study, 16S rRNA sequencing was used to analyze the effects of long-term chewing of betel nut on the gut microbiota of Hainanese. The BNC group consisted of individuals who chewed betel nuts daily for at least one year and excluded those who chewed betel nut intermittently. The results showed that chewing betel nut for a long time had a profound influence on the diversity and composition of gut microbiota.

A recent study showed similar overall community-level diversity in the guts of males and females, whereas the core species was significantly influenced by gender [16]. The evidence showed that the differences between gut microbiota of males and females could affect the susceptibility of sex-specific diseases [17]. In the present study, the α-diversity was higher for females than males, especially in the BNC group (P<0.05). Therefore, the results of our study verified the difference in gut microbiota between males and females and showed that gender interfered with the influence of long-term BNC on intestinal microflora.

Studies have suggested that changes in bacterial phyla are an essential factor in regulating host metabolism. Compared to healthy individuals, a decrease in the Firmicutes-to-Bacteroidetes ratio was found in patients with obesity [18], NAFLD [19], type 1 diabetes [20], and type 2 diabetes [21]. Bacteroidetes can synthesize many carbohydrate-active enzymes and produce vital metabolite short-chain fatty acids (SCFAs) [15]. SCFAs can be used as energy sources and critical small molecules to regulate host gene expression and inflammation [22]. Moreover, an increase in the abundance of Actinobacteria has usually been associated with diseases, such as inflammatory bowel disease [23], mental disorders [24, 25], type 2 diabetes [26], and epilepsy [27]. Cyanobacteria play a role in neurodegenerative diseases by producing a variety of toxic metabolites, such as β-methylamino-1-alanine, 2,4-aminobutyric acid, and N-2-aminoethylglycine [28]. In this study, the abundance of Firmicutes was increased and the abundance of Bacteroidetes was decreased in the BNC group compared to the Ctr group. The Firmicutes-to-Bacteroidetes ratio was significantly increased in the BNC group compared to the Ctr group. In addition, the abundances of Cyanobacteria and Actinobacteria were significantly increased in the BNC_Male group compared to the Ctr group. These results suggest that energy intake control could produce beneficial effects on BNC individuals by modulating the Firmicutes-to-Bacteroidetes ratio, while promoting the growth of disease-related microbial phyla.

Studies have shown that reduced Coriobacteriaceae abundance is linked to T2DM [29] and Parkinson’s disease [30]. Probiotic and prebiotic treatments have been shown to increase the relative abundance of Coriobacteriaceae [31, 32]. Increased abundances of Aerococcaceae, Neisseriaceae, Moraxellaceae, Porphyromonadaceae, and Planococcaceae have been reported for multiple diseases, such as rheumatoid arthritis [33], enteritis [34, 35], end-stage renal disease [36], cholelithiasis [37] and constipation [38]. In addition, in previous studies, an increased abundance of Streptococcaceae has been associated with diseases, such as kidney disease [39], atrophic gastritis [40], and depression [41]. Increased abundance of Micrococcaceae, Xanthomonadaceae, Coxiellaceae, Nocardioidaceae, Rhodobacteraceae, and Succinivibrionaceae has been associated with diseases, such as breast cancer [42], obesity [43, 44], asthma [45, 46], and cholangiocarcinoma [47]. In the present study, the abundance of Coriobacteriaceae was significantly increased in the BNC and BNC_Male groups compared to the Ctr group. Aerococcaceae, Neisseriaceae, Moraxellaceae, Porphyromonadaceae, and Planococcaceae were decreased in the BNC, BNC_Male, and BNC_Female groups compared to the Ctr group. The abundance of Streptococcaceae was significantly increased in both the BNC and BNC_Female groups compared to the Ctr group. The abundances of Micrococcaceae, Xanthomonadaceae, Coxiellaceae, Nocardioidaceae, Rhodobacteraceae, and Succinivibrionaceae were increased in the BNC, BNC_Male, and BNC_Female groups compared to the Ctr group. These results suggest that BNC has two sided effects on gut microbiota. That is, BNC can increase the abundance of potentially beneficial microbes and decrease that of disease-related microbes but can also increase the abundance of disease-related microbes.

Microbial function prediction suggested that BNC can inhibit PWY-5676, PWY-5677, and CODH-PWY. A previous study implicated PWY-5676 and PWY-5677 in the fermentation of ethanol, acetate, and succinate to butyrate, whereas CODH-PWY was implicated in the fermentation of acetyl-CoA to acetate [48, 49]. SCFAs, including butyrate and acetate, are mainly produced by the gut microbiome [50]. SCFAs have been estimated to provide 60–70% of the energy required by colon epithelial cells [51]. Studies have shown that butyrate is involved in regulating intestinal barrier function and immune and inflammatory responses [51, 52]. Butyrate can have beneficial effects on blood lipid levels, diabetes, and body weight [53]. However, some studies suggest that obese people have higher levels of SCFAs because SCFAs can provide a considerable quantity of energy [54, 55]. Therefore, BNC could affect host health by regulating metabolites in gut microbiota.

In this study, individuals were divided into youth (18–30 years old group, BNC: Ctr = 14:10), middle-age (31–45 years old group, BNC: Ctr = 13:11), Middle_elderly age (46–60 years old Group, BNC: Ctr = 10:13). BNC did not affect α-diversity in all age groups. Meanwhile, metastats analysis (using q-value<0.05 as the threshold) could not find significant differences in bacteria at the phylum, family, and genus level (S4 Table). In addition, there had no significant difference in α-diversity among age groups. Studies had shown that the diversity and composition of the gut microbiota changed with age [56, 57]. Therefore, more individuals should be included in future research to consider the impact of age and diseases on gut microbiota.

## 5. Conclusion

In summary, BNC may decrease microbial α-diversity. BNC was found to significantly affect the microbial phyla level, including increasing the Firmicutes-to-Bacteroidetes ratio compared to that of a control group. At the family level, BNC can increase potentially beneficial microbes and reduce disease-related microbes in the host. BNC was also found to increase the abundance of some disease-related microbes. Functional prediction showed that the metabolism of multiple substances (carbohydrates, vitamins, lipids, and amino acids) was significantly affected by BNC, leading to alteration of metabolites and further affecting host health. Therefore, investigating gut microbiota to analyze the possible impact of BNC on human health showed that the role of BNC is bidirectional. Further research on betel nut is warranted, and the beneficial components should be separated for subsequent development.

## Supporting information

S1 Table Bacteria that changed significantly in the MFA group. (XLSX)

S2 Table Bacteria that changed significantly in the FA group. (XLSX)

S3 Table Bacteria that changed significantly in the MA group. (XLSX)

S4 Table Bacteria that changed significantly in the different age groups. (XLSX)
